# Supplementary material for: Characterizing the genetic basis of trait evolution in the Mexican cavefish
Source: Evol Dev. 2022 Aug 4;24(5):131–44. doi: 10.1111/ede.12412 (PMC9786752; doi:10.1111/ede.12412)
Supplement: Supplementary file 1 — Supplementary information. [file EDE-24--s001.docx]

**Supplemental Figure Legends**

**Supplementary Figure 1: Landmarks used for morphometric analysis.** Each line depicts a measurement used for morphometric analysis in surface fish (top) and Pachón cavefish (bottom). Both dorsal (right) and side views were imaged for each individual fish and used for quantification.

**Supplementary Figure 2: Quantification of anatomical differences between surface fish and Pachón cavefish. A)** Height is significantly greater in cavefish compared to surface fish (t-test: t_59_=6.710, *P*<0.0001). **B)** Head length is significantly greater in cavefish compared to surface fish (t-test: t_59_=14.65, *P*<0.0001). **C)** Head width is significantly greater in cavefish compared to surface fish (t-test: t_59_=15.37, *P*<0.0001) **D**) Dorsal eye size is significantly reduced in cavefish compared to surface (t-test: t_59_=36.49, *P*<0.0001). **E**) There is no difference in average fin length between surface fish and cavefish (t-test: t_59_=1.422, *P*<0.1603). For each trait, the median (center line) as well as 25^th^ and 75^th^ percentiles (dotted lines) are shown. Circles represent values from individual fish. *** denotes P<0.001.

**Supplementary Figure 3: Quantification of normalized anatomical differences between surface fish and Pachón cavefish. A)** Normalized jaw width is significantly greater in cavefish compared to surface fish (t-test: t_59_=11.26, *P*<0.0001). **B)** Normalized eye size is significantly reduced in cavefish compared to surface fish (t-test: t_59_=49.76, *P*<0.0001). **C)** Normalized height is significantly reduced in cavefish compared to surface (t-test: t_59_=6.971, *P*<0.0001). **D)** Normalized head length is significantly greater in cavefish compared to surface (t-test: t_59_=9.817, *P*<0.0001). **E)** Normalized head width is significantly greater in cavefish compared to surface fish (t-test: t_59_=7.634, *P*<0.0001). **F)** Normalized dorsal eye size is significantly reduced in cavefish compared to surface (t-test: t_59_=62.14, *P*<0.0001). **G)** Normalized average fin length is significant reduced in cavefish compared to surface (t-test: t_59_=6.563, *P*<0.0001). For each trait, the median (center line) as well as 25^th^ and 75^th^ percentiles (dotted lines) are shown. Circles represent values from individual fish. *** denotes P<0.001.

**Supplementary Figure 4: Pairwise correlations between traits in surface and cave fish.** For each pairwise comparison, R^2^ values are shown. **A-B)** Heat map of the correlations between morphological traits in **(A)** cave and **(B)** surface fish. **C-D)** Heat map of the correlations between behavioral traits in **(C)** cave and **(D)** surface fish. **E-F)** Heat map of the correlations between morphological and behavioral traits in **(E)** cave and **(F)** surface fish.

**Supplementary Figure 5: Quantification of anatomical differences between pigmented and albino F2 hybrid offspring. A)** Length does not differ between pigmented and albino individuals (t-test: t_121_=0.3415, *P*<0.7334). **B)** Jaw width does not differ between pigmented and albino individuals (t-test: t_121_=0.7447, *P*<0.4579). **C**) Height does not differ between pigmented and albino individuals (t-test: t_121_=0.8388, *P*<0.4032). **D)** Head length does not differ between pigmented and albino individuals (t-test: t_121_=0.3090, *P*<0.7578). **E**) Head width does not differ between pigmented and albino individuals (t-test: t_121_=1.265, *P*<0.2084). **F**) Dorsal eye size does not differ between pigmented and albino individuals (t-test: t_121_=1.780, *P*<0.0775). **G**) Average fin length does not differ between pigmented and albino individuals (t-test: t_121_=0.8878, *P*<0.3764). **H**) Normalized eye size is significantly reduced in albino compared to pigmented hybrid offspring (t-test: t_121_ =2.107, *P*<0.0372). For each trait, the median (center line) as well as 25^th^ and 75^th^ percentiles (dotted lines) are shown. Albino individuals are depicted as red squares, while pigmented individuals are depicted as black circles.

**Supplementary Figure 6: Quantification of behavioral differences between pigmented and albino F2 hybrid offspring. A**) Strike angle does not differ between pigmented and albino individuals (t-test: t_116_=0.0488, *P*<0.9611). **B)** Strike distance does not differ between pigmented and albino individuals (t-test: t_116_=0.0182, *P*<0.9855). **C)** Angular speed does not differ between pigmented and albino individuals (t-test: t_45_=0.0852, *P*<0.9325). **D)** Peak angle does not differ between pigmented and albino individuals (t-test: t_45_=1.354, *P*<0.1826). For each trait, the median (center line) as well as 25^th^ and 75^th^ percentiles (dotted lines) are shown. Albino individuals are depicted as red squares, while pigmented individuals are depicted as black circles.
