# Supplementary material for: Characterizing the genetic basis of trait evolution in the Mexican cavefish
Source: Evol Dev. 2022 Aug 4;24(5):131–44. doi: 10.1111/ede.12412 (PMC9786752; doi:10.1111/ede.12412)

Supplementary Figure 1

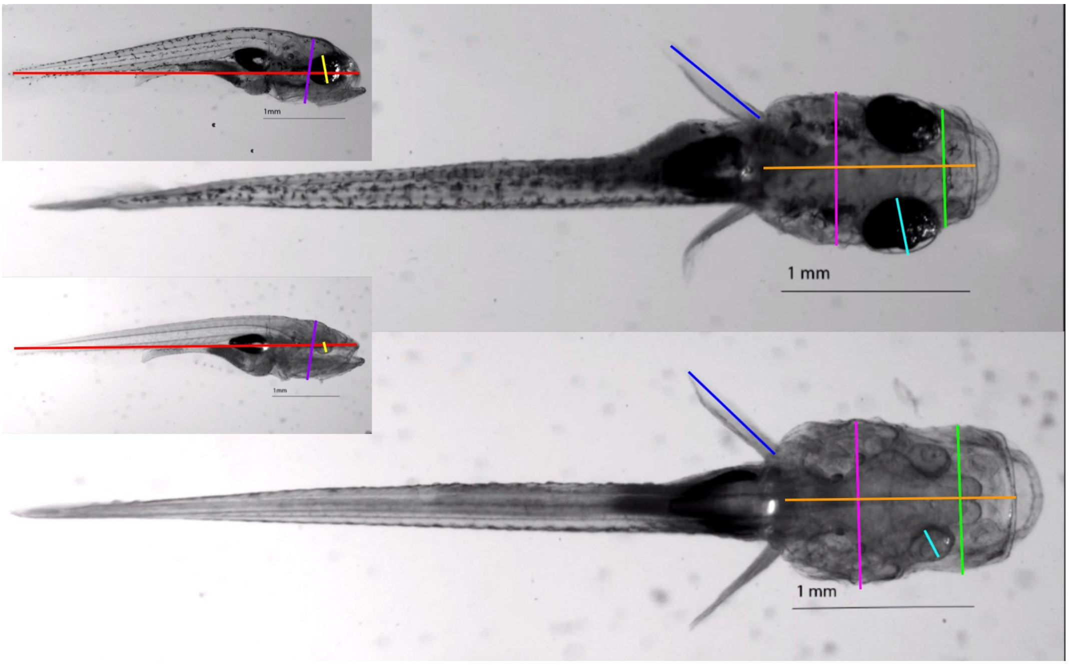

Supplementary Figure 2

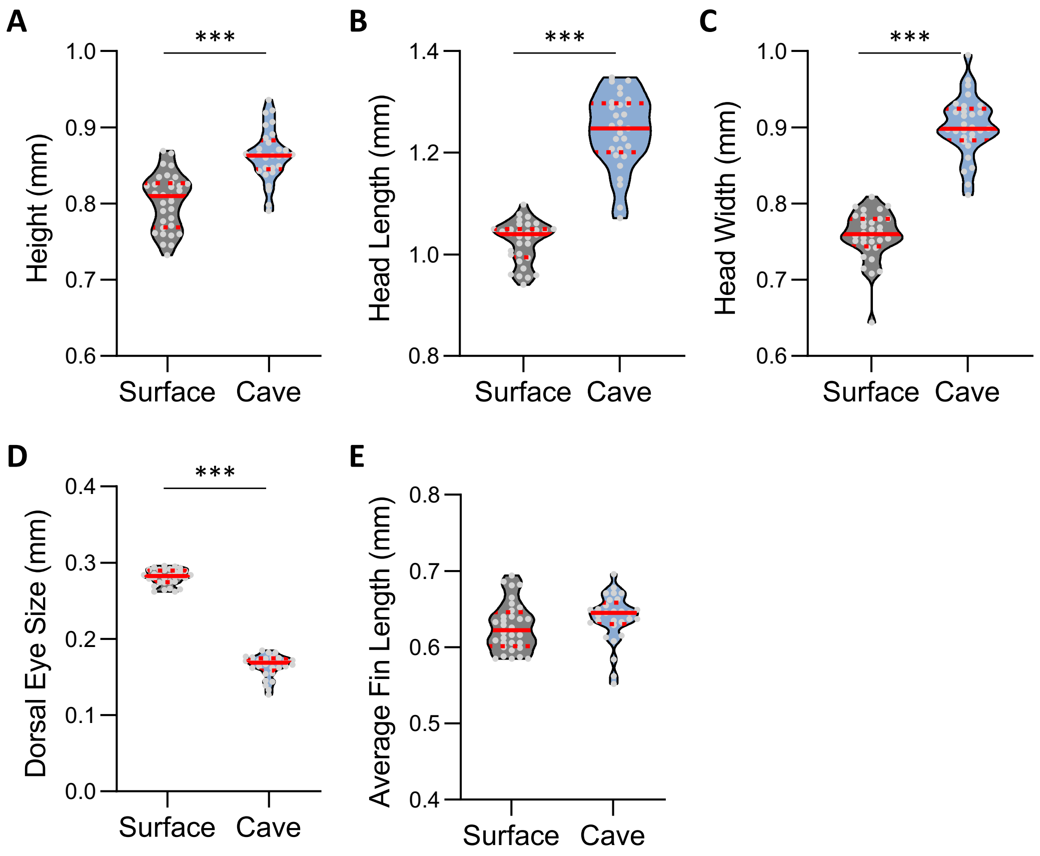

Supplementary Figure 3

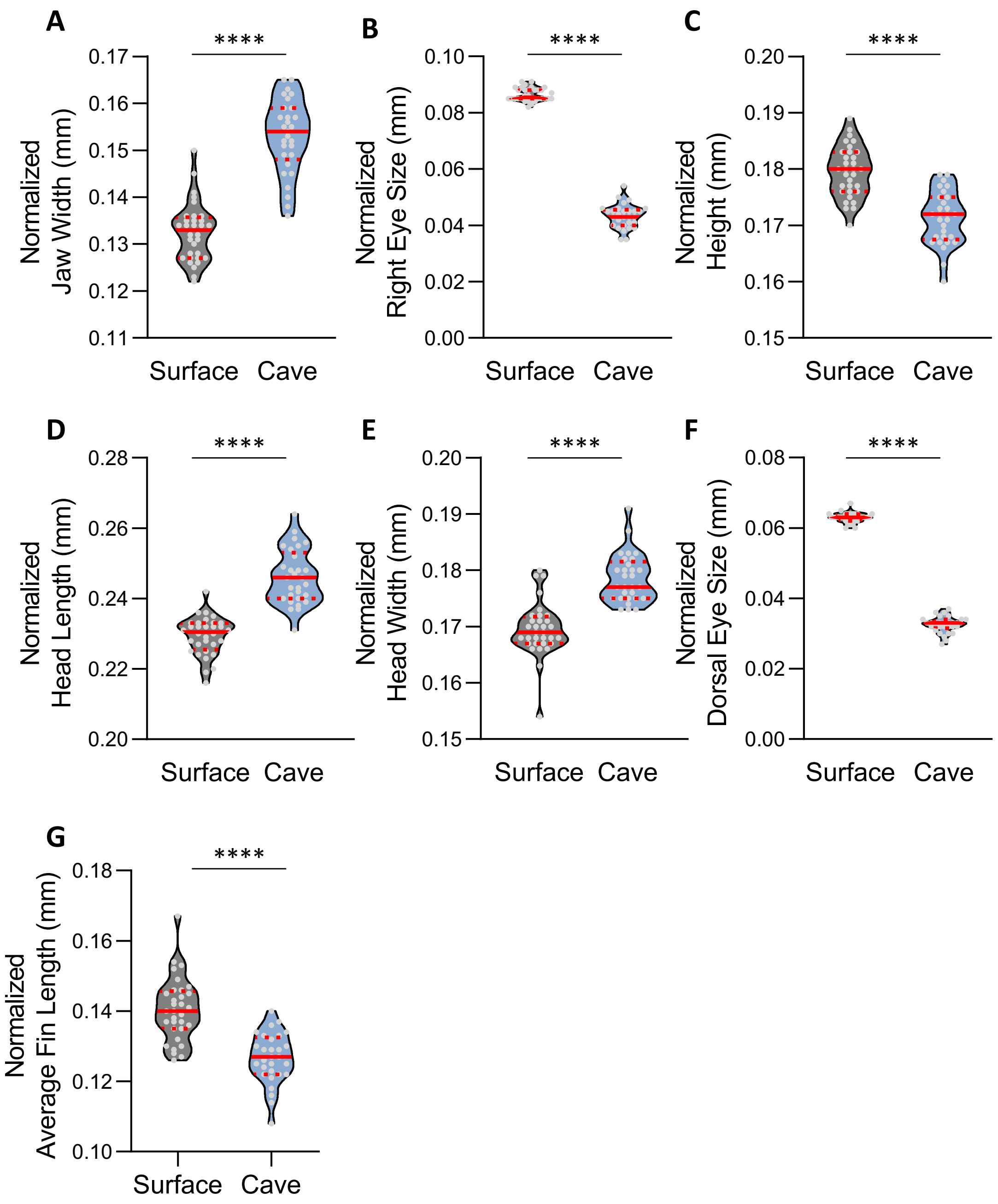

Supplementary Figure 4

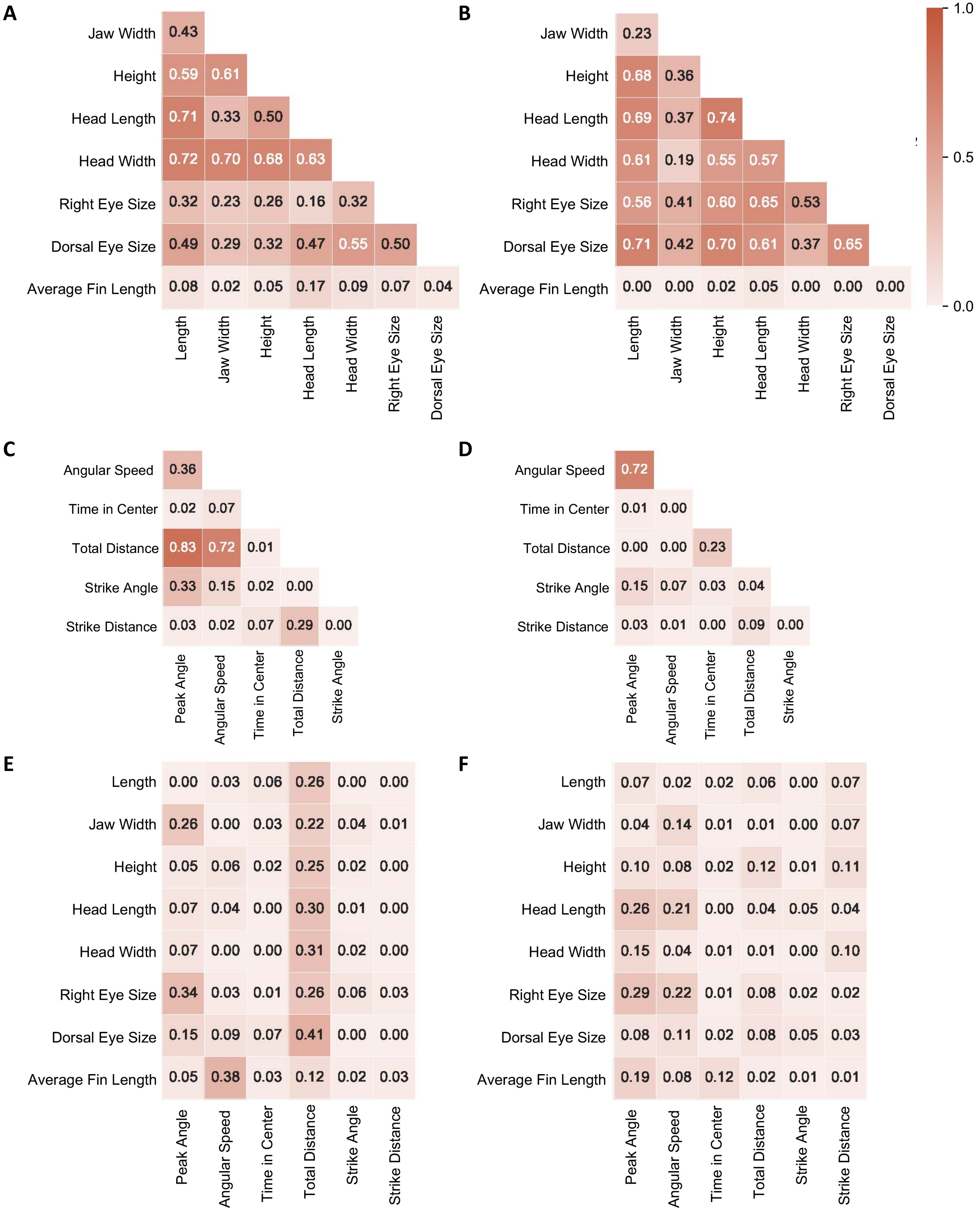

Supplementary Figure 5

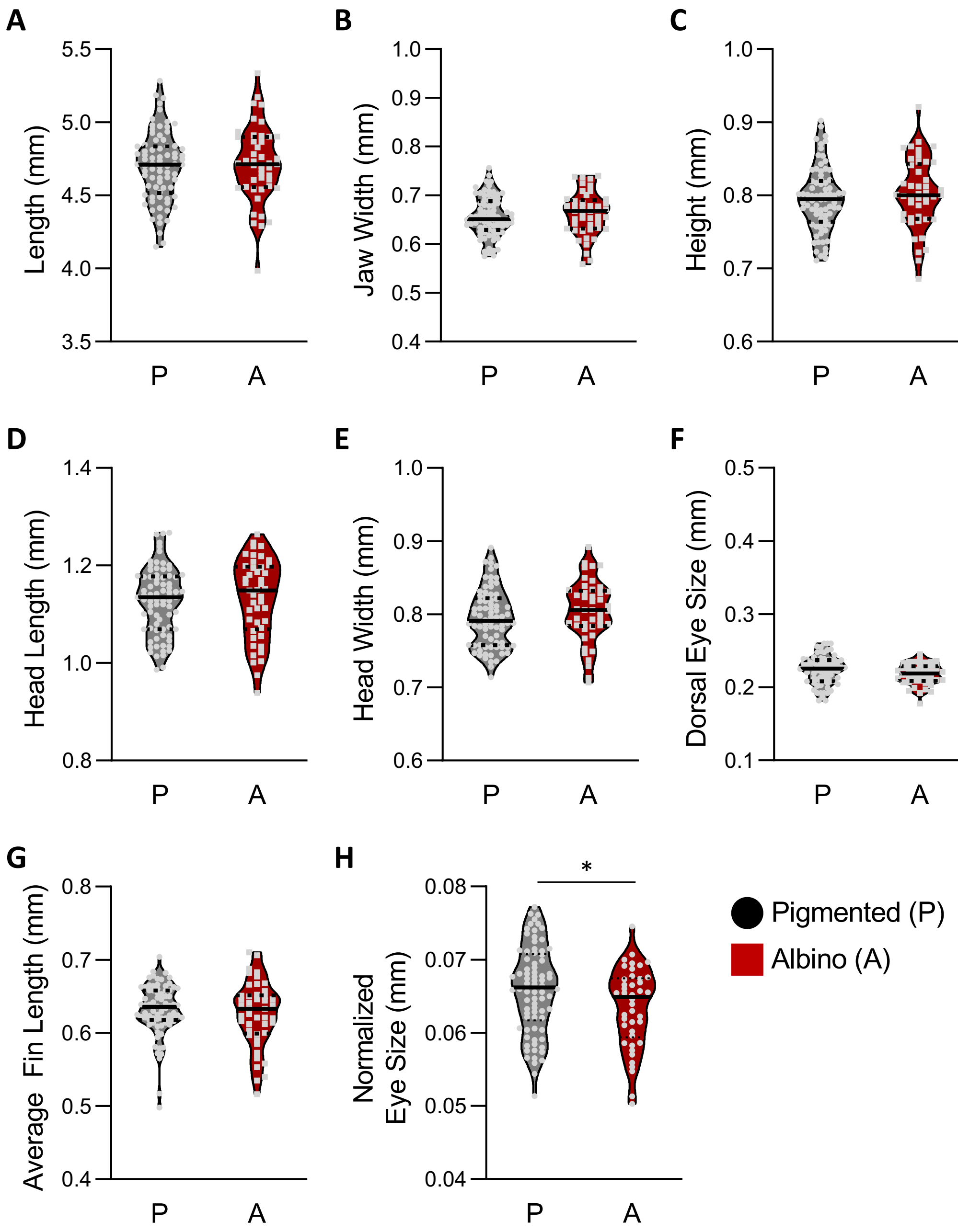

Supplementary Figure 6

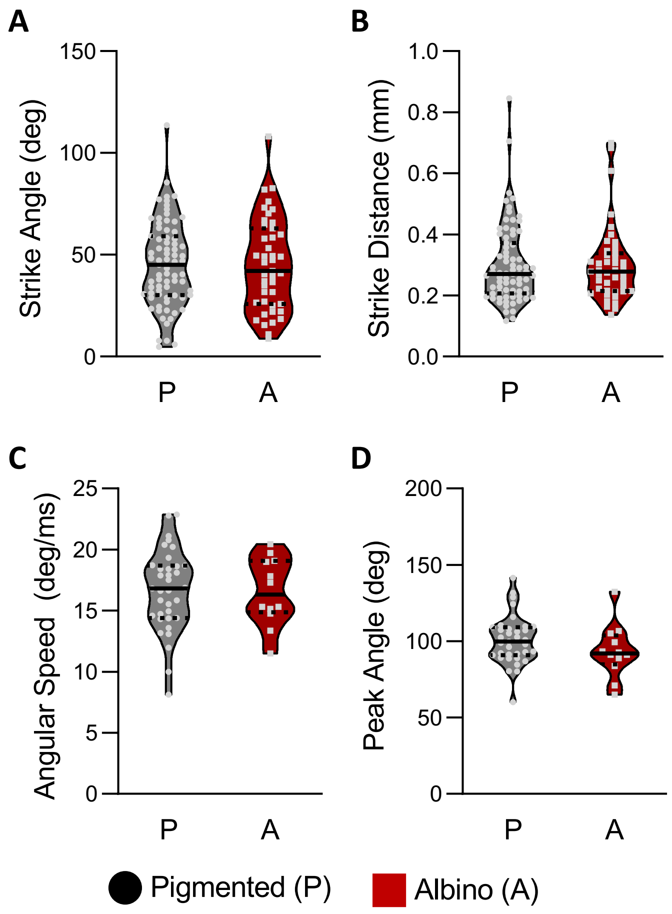

Supplement: Supplementary file 3 — Supplementary information. [file EDE-24--s003.pdf]
